# Probing the Influence of Paternal Diet on Offspring Neuroanatomy With Mouse MRI

**DOI:** 10.1002/brb3.71195

**Published:** 2026-01-28

**Authors:** Emma G. W. McKnight, Gail Lee, Cheryl Chong, Jane A. Foster, Tie‐yuan Zhang, Jason P. Lerch, Brian J. Nieman, Mark R. Palmert

**Affiliations:** ^1^ Mouse Imaging Centre The Hospital for Sick Children Toronto Ontario Canada; ^2^ Translational Medicine The Hospital for Sick Children Toronto Ontario Canada; ^3^ Department of Medical Biophysics University of Toronto Toronto Ontario Canada; ^4^ Department of Biochemistry University of Waterloo Waterloo Ontario Canada; ^5^ Department of Psychiatry and Behavioural Neurosciences McMaster University Hamilton Ontario Canada; ^6^ Center for Depression Research and Clinical Care, Department of Psychiatry UT Southwestern Medical Center Dallas Texas USA; ^7^ Douglas Mental Health University Institute, Department of Psychiatry McGill University Montréal Quebec Canada; ^8^ Wellcome Centre for Integrative Neuroimaging University of Oxford Oxford UK; ^9^ Department of Physiology The University of Toronto Toronto Ontario Canada; ^10^ Division of Endocrinology The Hospital for Sick Children Toronto Ontario Canada

**Keywords:** high‐fat/high‐simple sugar, MRI, neurodevelopment, paternal diet

## Abstract

**Purpose:**

Previous studies have established that parental consumption of a diet high in fat and simple sugar (HF/HSS) leads to long‐term effects on offspring brain development. However, most studies have focused on the effects of maternal diets or the combined effects of both parents’ diets. As literature suggests that fathers’ environmental factors can also impact offspring brain development, we aimed to explore the impact of isolated paternal consumption of an HF/HSS diet on offspring brain structure.

**Methods:**

C57Bl/6J male mice were acclimated to an HF/HSS diet for eight weeks prior to mating with females who consumed standard chow (control diet, CD). A matching paternal control group was fed the CD during the acclimation period. Throughout gestation and lactation all dams and offspring were fed the CD; all pups were weaned at postnatal day 21 (P21) and stayed on the CD. At P42 offspring brains were prepared for *ex vivo* magnetic resonance imaging (MRI). Brain MR images were then segmented for volumetric structural analysis.

**Results:**

HF/HSS‐fed sires gained more weight during acclimation than CD sires (*p* < 0.001). However, offspring weights at weaning (P21) and at endpoint (P42) were not significantly affected by paternal diet. Offspring brain morphology, as assessed by volume measurements of 185 brain structures, was not significantly affected by sire HF/HSS diet alone.

**Conclusion:**

While small structural changes cannot be ruled out, the results suggest that previously observed changes in offspring brain structure attributed to parental consumption of HF/HSS diet (selected to mimic some aspects of the human “Western Diet”) require maternal consumption.

## Graphical abstract

This study aimed to determine if paternal consumption of a high‐fat/high‐simple‐sugar (HF/HSS) diet prior to conception recapitulates the offspring brain structure volume changes seen with parental preconception consumption of an HF/HSS diet. Using C57Bl6/J mice and MRI, we established that there were no significant effects of paternal HF/HSS eating habits on their offspring's brain structure volumes despite significant effects on paternal weight.

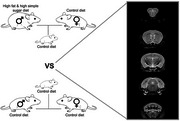

## 1 Introduction

It is well established that human consumption of a diet high in fat and simple sugar, which are components of what is often referred to as the “Western Diet” (WD), increases risk for a wide range of ailments. WD consumption is linked to overweightness and obesity (Cordova et al. 2021; Hall et al. 2019), type 2 diabetes (O'hearn et al. 2023), cardiovascular diseases (Valenzuela et al. 2023), and numerous cancers (Mehta et al. 2017; Papadimitriou et al. 2021). Consumption of WD prior to and during pregnancy can also induce long‐term effects for offspring. Combined maternal and paternal (i.e., parental) WD consumption is associated with increased risk of offspring being diagnosed with a neurodevelopmental disorder (NDD) such as attention deficit/hyperactivity disorder (ADHD) (Li et al. 2020) and autism spectrum disorder (ASD) (Suren et al. 2014). Other data indicate that parental impact on offspring health is not limited to specific diet patterns but also the ensuing metabolic changes. For example, regardless of diet, mothers being overweight or obese pre‐pregnancy is associated with increased risk of offspring being diagnosed with neuropsychiatric disorders with a neurodevelopmental basis, such as anxiety, depression, and schizophrenia (Kong et al. 2020; Rivera et al. 2015; Sarker et al. 2019). Taken together, these data indicate that parental diets that expose the offspring to these metabolic states may be a modifiable risk factor for offspring NDDs.

While it is intuitive to presume that maternal environmental influences have the greatest influence on offspring development, paternal environmental factors—independent of maternal exposures—may also affect offspring health. For example, in mice, paternal exposure to endocrine‐disrupting chemicals (EDCs) has been associated with decreased fertility (Adegoke et al. 2022) and increased anxiety and depression‐like behaviors in offspring (Fan et al. 2018), and paternal alcohol consumption has been linked to reduced offspring growth rates and increased ADHD‐like symptoms (Kim et al. 2014). Paternal diet alone has been connected to modified risks for offspring health and development in rodent models as well. Low paternal protein intake was reported to impair offspring cardiovascular and metabolic function (Morgan et al. 2020; Oke and Hardy 2017; Watkins and Sinclair 2014), disrupt fetal growth and skeletal development (Morgan et al. 2021; Watkins et al. 2017), and increase offspring risk for mammary and other solid tumors (Da Cruz et al. 2018). Positive impacts have also been observed: a paternal diet high in protein resulted in better offspring body composition, more balanced gut microbiota, and a reduced incidence of obesity and diabetes (Chleilat et al. 2021). These examples show that the paternal environment alone, including a modified diet, can influence offsprings’ health and development.

In previous work, we have used mouse models to demonstrate that various obesogenic diet formulations, when fed to dams and sires, can impact offspring neurodevelopment based on MRI measurements of brain structure volumes (Fernandes et al. 2021; Lee et al. 2025). Some of the observed brain changes were in regions linked to NDDs in humans, underscoring the need to understand the impact of these exposures more fully. Given the studies that demonstrate the impact of paternal environment on offspring phenotypes, we sought to determine the impact of paternal diet alone on offspring brain development. Thus, in this study, we limited experimental diet exposure to sires and investigated the potential impacts on offspring using MRI.

## 2 Materials and Methods

### 2.1 Animals and Experimental Diets

Five‐week‐old male and female C57Bl/6J mice were obtained from the in‐house breeding colony at The Centre for Phenogenomics (TCP; Toronto, Ontario, Canada). All mice were housed in a controlled environment on a 12/12 h light/dark cycle, with temperature maintained between 21–23°C and humidity levels between 40%–60%. Food and water were available ad libitum to all mice throughout the study. Diets included both a standard chow control diet (CD) and a high fat/high simple sugar diet (HF/HSS) selected to mimic some aspects of the human “Western Diet.” The CD contained 19.1% of kcals from protein, 67.9% from carbohydrates, and 13.0% from fat, with 120.0 g/kg of sucrose (TD08485, Inotiv, formerly Envigo, IN, USA). The HF/HSS diet contained 15.2% of kcal from protein, 42.7% from carbohydrates, and 42.0% from fat, with 341.46 g/kg of sucrose as a source of simple sugar (TD88137, Inotiv, IN, USA). Males were placed on either the CD or HF/HSS diet for an eight‐week acclimation period, during which food (HF/HSS or CD) was placed on the floor of the cage. Breeding females were fed the CD throughout the same eight‐week acclimation period. Following the acclimation period, male and nulliparous female mice (20 breeding pairs per diet) were paired for breeding for 5 days and provided with CD only. Pregnant female mice were then singly housed and continued to have access to CD throughout gestation and lactation. Litters were culled down to a maximum of six pups, with an even sex ratio whenever possible. Pups were weaned at postnatal day (P) 21 onto the CD and housed with their littermates of the same sex. Mice used as parents were only bred once to ensure a consistent exposure time to the experimental diet. Animal care and experimental procedures were approved by the TCP Animal Care Committee (AUP 25–0175H).

To evaluate the effect of the diets on weight of the sires, male mice were weighed every week of the eight‐week acclimation period. Offspring were weighed at weaning (P21) and at the end of the experimental period (P42). Both sire and offspring weights were examined with a t‐test to determine impact of diet.

### 2.2 Brain Sample Preparation

Fixed whole‐brain samples were prepared for MRI to assess the impact of paternal diet on the neuroanatomy of offspring: *n* = 23 M/24 F, 25 M/26 F (CD, HF/HSS diet, respectively). At P42 ± 1, offspring were deeply anesthetized with isoflurane until loss of pedal reflex. They were then intracardially perfused with a 30 mL solution of phosphate‐buffered saline (PBS) (311‐010 CL, Wisent, Saint‐Jean‐Baptiste, QC), 1 µL/mL heparin (heparin sodium, Fresenius Kabi Canada Ltd., Toronto, ON), and 2 mM ProHance (gadoteridol, Bracco Diagnostics Inc., Princeton, NJ). This was followed with a 30 mL fixative solution of PBS, 4% paraformaldehyde (PFA) (16% w/w, Electron Microscopy Sciences, Biolyst, Hatfield, PA), and 2 mM ProHance. Following perfusion, the head was removed from the body, and the extracranial tissue was removed, leaving the brains in the skulls. The skulls were stored overnight in the same fixative solution and then were stored in a storage solution of PBS, 2 mM ProHance, and 0.02% sodium azide (Fisher BioReagents, Thermo Fisher Scientific, Waltham, MA) for five months at 4°C (Cahill et al. 2012; de Guzman et al. 2016), after which they were imaged.

### 2.3 Image Acquisition

Brains were scanned with a 7‐Tesla 306‐mm horizontal bore magnet (BioSpec 70/30 USR, Bruker, Ettlingen, Germany) fitted with a custom‐made coil array that can scan up to 8 brains in parallel (modified from Dazai et al. 2011). Samples were scanned with a three‐dimensional T2‐weighted sequence with the following parameters: TR = 350 ms, 12 ms echo spacing, 6 echoes, TEeff = 30 ms, 630 × 504 × 504 matrix, 40 um isotropic resolution, and 4 effective averages; a total scan time of 13 h (Spencer Noakes et al. 2017).

### 2.4 Image Registration and Analysis

Images were registered together using the Pydpiper toolkit (Friedel et al. 2014), which aligns all brain images into a consensus average space. Each brain image was also automatically segmented into 185 bilateral structures using the MAGeT Brain segmentation algorithm (Chakravarty et al. 2013) using the DSURQE atlas (with contributions from several groups: Dorr et al. 2008; Qiu et al. 2018; Richards et al. 2011; Steadman et al. 2014; Ullmann et al. 2013; accessible at https://tinyurl.com/MICeatlas).

All statistical analyses were performed using RMINC (Lerch et al. 2017) in R (version 3.6.3). To determine the effect of paternal diet on offspring neuroanatomy, the relative structure volumes (% of total brain volume) were fit using a linear mixed effects (LME) model (Bates et al. 2015) that included fixed‐effect coefficients for paternal diet, offspring sex, and their interaction, as well as a random‐effect coefficient to account for litter effects. P‐values were calculated based on the Satterthwaite approximation (Kuznetsova et al. 2020). The false discovery rate (FDR) was employed to correct for multiple comparisons (Benjamini and Hochberg 1995). Volumetric data and MRI data can be found at https://doi.org/10.6084/m9.figshare.30356281.

### 2.5 Power Analysis

Motivated by initial findings, we wanted to estimate the smallest possible brain volume changes likely to have been captured with our sample design, which we refer to as the minimum detectable volume change (MDVC). Using previously acquired data, we created simulated brain volume changes for all 185 brain structures based on a range of percent volume changes. From this simulated data, we set the MDVC to be the level of percent change for each structure that exhibited a statistically significant (*p* < 0.001) change in at least 80% of simulations.

More specifically, to estimate the MDVC given the number of mice and anticipated variance by structure, we performed a data simulation based on a separate dataset from P42 offspring where both members of the mating pair had been fed either a CD or HF/HSS diet (parental diet exposure). From this parental diet data, for each structure, we extracted the mean relative volume by offspring sex, the standard deviation of litter effects, and the standard deviation of residual errors. Using these measures, we generated simulated datasets (3000 iterations) for each structure individually with percent volume changes of 0, 0.5, 1.0, 1.5, 2.0, 3.0, 5.0, 7.0, 11.0, 15.0, 20.0, and 40.0% (modelling induced changes by parental HF/HSS consumption).

Using the previously described linear mixed effects model, we compared the simulated effects of a parental HF/HSS diet to the CD‐fed reference data and recorded the level of significance (p value) for all iterations. We then recorded the frequency of significance, which was below our chosen p‐value threshold of 0.001. This p‐value was selected as corresponding to a relaxed FDR threshold of roughly 20% in the context of no significant findings. Linear interpolation was used to estimate MDVC with 80% power between the simulated volume changes, which we report as the nominal MDVC for our experiment (Table S1).

## 3 Results

### 3.1 HF/HSS Diet Induced Weight Gain in Fathers, but Not in Offspring Consuming CD

**FIGURE 1:**
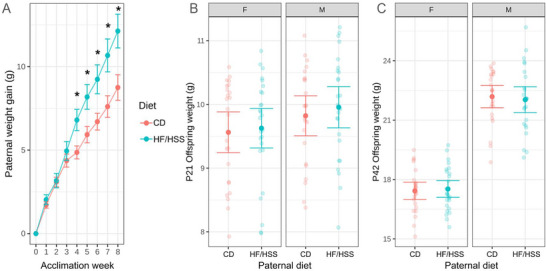
Paternal HFSS diet consumption induced weight gain in sires but not in offspring. (A) The average weight gained (with 95% confidence interval) by sires over the 8‐week acclimation period was greater in HF/HSS sires than in CD sires. Weight gained was determined by subtracting the weight of the sires at the start of their experimental diet (*, *p* < 0.05). (B) Absolute weight of the female and male offspring at weaning (P21) was not significantly different based on paternal diet (F, female; M, male). (C) Absolute weight of the offspring at the end of the experimental timeline (P42) was not significantly different based on diet exposure.

To verify that HF/HSS diet consumption over the eight‐week acclimation period induced changes in sires, mice were weighed every week while on the diet. In reference to their starting weight (at week 0), the weight gain of HF/HSS sires was significantly higher than CD sires (*p* < 0.001) by the fourth week of acclimation and continued to be higher through the end of the acclimation period (Figure 1A). On average, HF/HSS‐fed males gained 36% more than CD after the 8‐week acclimation.

We also evaluated the effect of paternal diet on offspring weights at weaning (P21), equivalent to early childhood in humans, and at the end of the experiment (P42), equivalent to late adolescence. At both timepoints the average absolute weights of the pups, separated by sex, were not affected by paternal diet (*p* > 0.5; Figure 1B‐C).

### 3.2 Paternal HF/HSS Diet Consumption Had No Impact on Offspring Brain Structure

**FIGURE 2:**
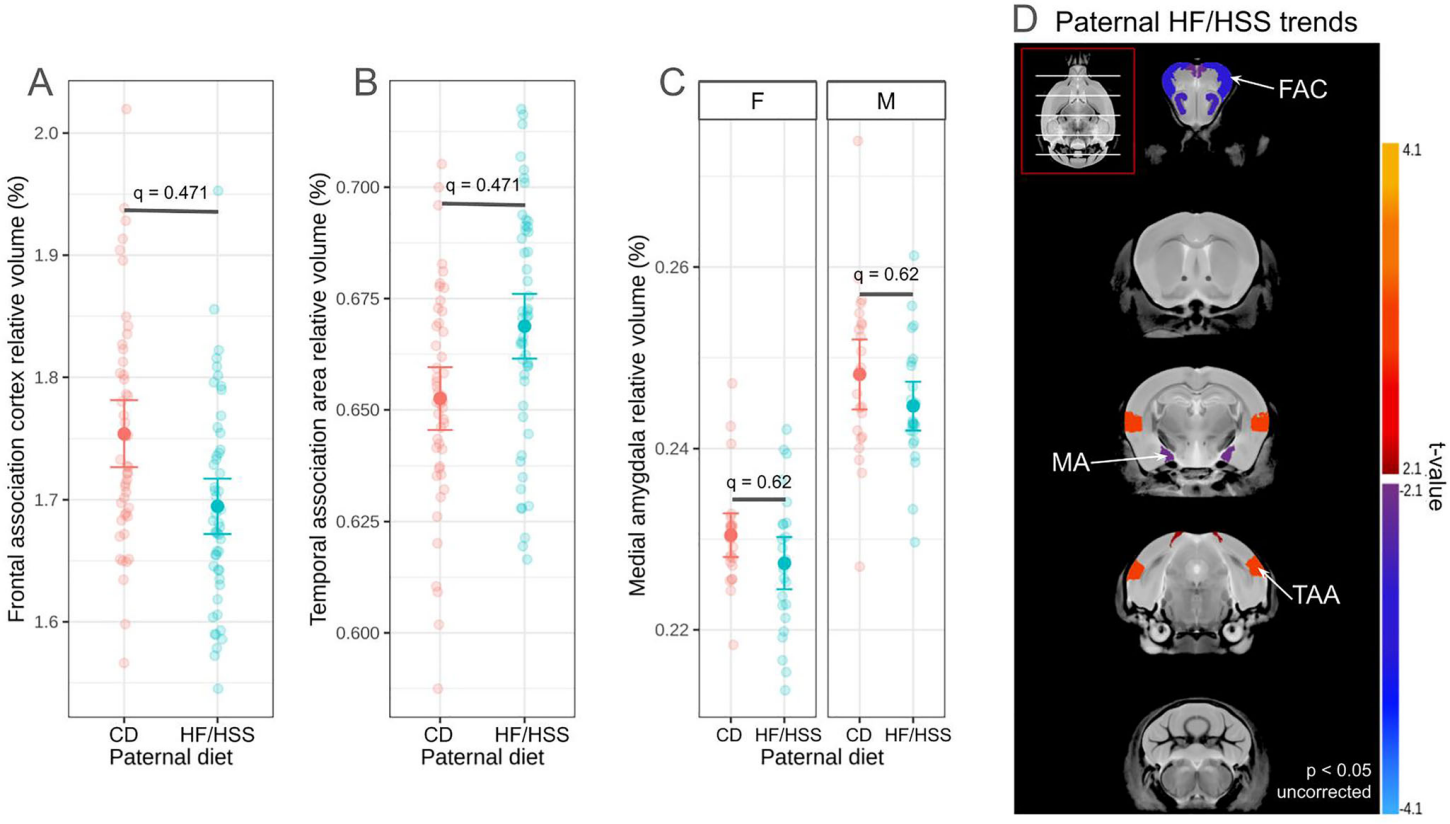
Paternal HF/HSS consumption induced no statistically significant changes in offspring brain structure volumes, but some subthreshold estimates are visualized. (A‐B) Individual plots show relative volumes (% of total brain volume) of select structures, none of which were considered significant after FDR correction. (C) Plot of the relative volume of the medial amygdala separated by sex. No statistical significance after FDR correction between diet groups was detected. However, a statistically significant (*p* < 0.0001) difference was found between sexes, as expected. (D) Brain structures that have a paternal HF/HSS effect at uncorrected *p* < 0.05 are colored. Scales represent degree of change; negative t‐values (blue‐purple scale) represent the offspring from HF/HSS sires having smaller structure volume in comparison to the CD offspring. All data were collected at endpoint, offspring age P42. FAC: Frontal association cortex, MA: medial amygdala, TAA: temporal association area.

Offspring were sacrificed at postnatal day 42 to determine the impacts of paternal diet on neuroanatomy at a timepoint equivalent to late adolescence (*n* = 23 M/24 F, 25 M/26 F [CD, HF/HSS diet]). After applying a 10% FDR correction for multiple comparisons, no statistically significant volume changes were identified. At a relaxed *p*‐value < 0.05, uncorrected for multiple tests, the relative volumes of twelve structures showed a trend of altered volume due to paternal HF/HSSS diet consumption independent of sex (Figure 2A). These structures included the frontal association cortex (‐3.32%, uncorrected *p* = 0.006), medial amygdala (‐1.74%, uncorrected *p* = 0.03), and the temporal association area (2.52%, uncorrected *p* = 0.009) (Figure 2B‐D). Note that at this threshold, 9–10 structures would be expected to be identified by chance.

**FIGURE 3:**
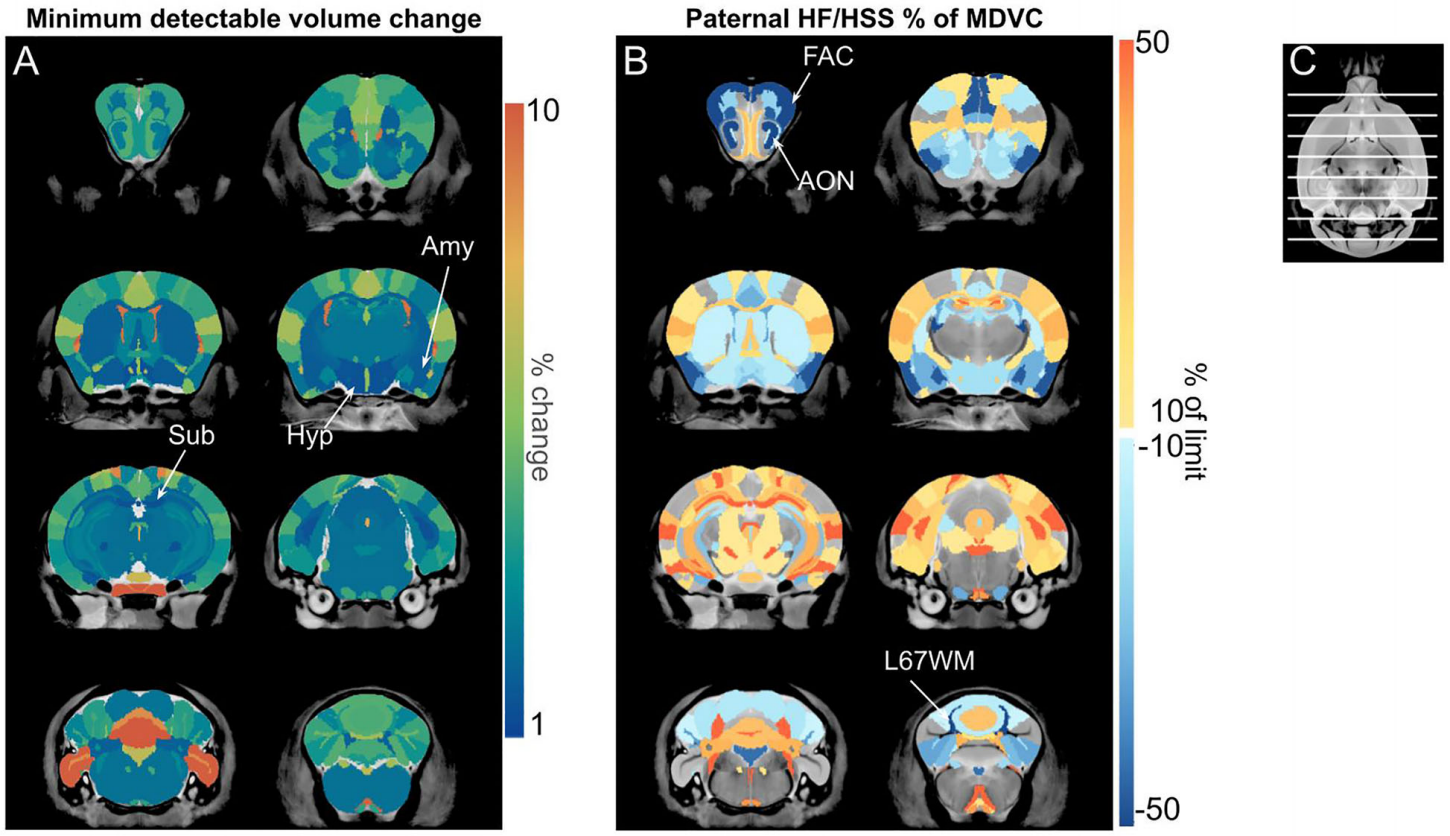
Examination of minimum detectable volume changes due to paternal HF/HSS. (A) Minimum detectable volume changes (MDVC) as % change for each structure is displayed, with the color scale limited to 1%–10% (some MDVC lie outside these limits). Sub, Amy, and Hyp have the smallest MDVC (1.43%, 1.73%, and 1.43%, respectively). (B) Proportion of MDVC seen to be induced by paternal HF/HSS consumption, as measured by this study. Values shown range from 10%–50% of structure‐specific MDVC (uncolored regions indicate values outside of this range). Positive changes (greater volume in HF/HSS offspring) are shown in the warm (yellow to red) color palette, and negative changes (smaller volume in HF/HSS offspring) are shown in the cool (light blue to dark blue) color palette. FAC, AON, and L67WM are the structures that achieve the highest% of their associated MDVC (81.2%, 67.5%, and 70.3%, respectively) (C) The slice indicator shows the location of the coronal slices for panels A and B. Sub: subiculum; Hyp: hypothalamus; Amy: amygdala; FAC: frontal association cortex; AON: anterior olfactory nucleus; L67WM: lobules 6 and 7 white matter.

Given our negative results, we sought to examine the limits of volume changes that might have been present but missed in our analysis (that is, type 2 error). To do this, we performed a simulation to estimate the minimum detectable volume change (MDVC) for each structure of the brain. Lower detection limits, that is, lower MDVC, indicate a smaller relative variance and a corresponding greater sensitivity for our analysis to have detected change. The simulation analysis showed that the MDVC ranged from a 1.4% change for the subiculum and hypothalamus to a 20.0% change for lobules 1&2 white matter in the cerebellum (Figure 3A); note that this change in volume represents a range of 0.5 % change in linear dimension. Maps indicating the estimated MDVC across the whole brain are provided in Figure 3A, providing an estimated limit to volume changes induced by paternal diet, if present. For visualization, we also computed the model‐estimated paternal volume changes as a percentage of MDVC (Figure 3B; Table S1).

## 4 Discussion

In this study, we employed structural MRI to assess the impact of a high‐fat, high‐simple‐sugar paternal diet on the offspring brain structure. The diet was chosen to model aspects of the commonly consumed human “Western Diet.” Although HF/HSS diet consumption showed the expected effects on paternal weight, we found no statistically significant effect on offspring brain structure. In contrast, comparable experiments in which both parents received the HF/HSS diet had shown significant offspring volume changes (Fernandes et al. 2021; Lee et al. 2025). Taken together, these findings suggest that maternal HF/HSS consumption leads to the majority of findings previously documented with both sire and dam diet exposure.

Of course, we cannot rule out the possibility that small structural changes induced by paternal diet would be detectable using larger sample sizes or that combined paternal and maternal diet exposure has a greater effect than a maternal‐only diet. We estimated the minimum detectable volume change for each structure, which suggested detection limits as low as 1.8% in many regions, indicating that if paternal diet induced volume changes, these are likely quite small. Notably, however, our measurements are less sensitive to volume change in cortical and cerebellar structures as compared to subcortical ones. While past studies have established that mouse models exhibiting behavioral changes generally also exhibit structural ones (Lerch et al. 2011; Nieman et al. 2007), it is possible that the paternal HF/HSS diet impacted offspring brains in ways that structural MRI cannot detect. Further studies could explore alternative neuroimaging approaches to assess different tissue properties (e.g., diffusion tensor imaging), immunohistochemistry to assess the levels of inflammation markers, or single‐cell transcriptomics to evaluate alterations in mRNA expression profiles.

While we found no impact of paternal HF/HSS diet on brain structure using MRI in this study, the hypothesis that paternal diet could lead to discernible changes was important to test. Because it has been demonstrated by others that parental/maternal diet and metabolic state are potential modifiable risks for neurodevelopmental disorders among offspring, it is important to understand the various contributors and how they can impact offspring development. Other studies evaluating the impact of a high‐fat sire diet and sire obesity have shown that offspring have delayed physical development (Binder et al. 2012), dysregulated metabolism (Park et al. 2018), and increased risk of kidney disease (Chowdhury et al. 2016). Other studies have also shown that paternal diets high in sugar and/or fats (Bodden et al. 2022; Freire et al. 2024), methyl donors (Ryan et al. 2018), or enriched omega‐3 fatty acids (Li et al. 2022) are able to modify offspring behavior and gene expression across the brain. If these changes were present in our mice, they did not substantially influence neurodevelopment in our model.

One potential environmental factor that could influence our findings is altered maternal care. It has been observed that dams mating with males who differ from standard weight (i.e., food‐restricted or obese) increase their care and attention to offspring, as measured by time spent licking and grooming (Mashoodh et al. 2018; Bodden et al. 2022), which appears to mitigate some of the effects of paternal diet. To isolate the paternal diet effects from the potential changes in maternal behavior induced by paternal diet, embryo transfer experiments would need to be performed, as in (Korgan et al. 2018).

The major strength of this study, beyond investigating the poorly studied realm of paternal dietary influences on offspring neurodevelopment, is the whole‐brain context provided by neuroimaging. By using *ex vivo* MRI, we were able to characterize the brain structure volume changes of 183 brain structures covering the whole brain in nearly 100 mice. We were also able to characterize the inherent limits of our study by determining the MDVC for each brain structure. This map should be of relevance to other simple two‐group C57BL/6J mouse MRI studies of similar design and size.

## 5 Conclusion

Previous studies have examined the effect of combined maternal and paternal diet on offspring brain structure. Here we investigated the impact of isolated paternal preconception HF/HSS diet consumption on offspring brain structure. This diet was selected to model the high fat and high simple sugar components of the human “Western Diet.” We demonstrated that HF/HSS consumption by fathers alone did not induce statistically significant changes in offspring brain structure volumes. Thus, it seems reasonable to conclude that previously observed impacts of parental diet on brain structure (Fernandes et al. 2021; Lee et al. 2025) derived principally from maternal and not from paternal influences (though combined maternal/paternal effects are, of course, a possibility). Thus, maternal diet consumption is required to affect offspring brain morphology development in this model.

## Author Contributions

EGWM, GL, BJN and MRP contributed to the conceptualization and metholodological design of the study. EGWM, GL, and CC conducted the investigation and data curation. EGWM performed the formal data analysis, visualization and writing of the original manuscript draft. BJN and MRP provided primary supervision and project administration. JAF, TYZ, JPL, BJN and MRP acquired funding for the project. All authors participated in writing through review and editing of the manuscript.

## Ethics Statement

All animal protocols were overseen and approved by the Centre for Phenogenomics animal care committee (AUP 25‐0175H).

## Conflicts of Interest

The authors declare no conflicts of interest.

## Supporting information




**Supplementary Table S1**: brb371195‐sup‐0001‐TableS1.xlsx

## Data Availability

Data can be found at doi.org/10.6084/m9.figshare.30356281.
